# Ceria Nanoparticles Alleviated Osteoarthritis through Attenuating Senescence and Senescence-Associated Secretory Phenotype in Synoviocytes

**DOI:** 10.3390/ijms24055056

**Published:** 2023-03-06

**Authors:** Xunshan Ren, Huangming Zhuang, Fuze Jiang, Yuelong Zhang, Panghu Zhou

**Affiliations:** Department of Orthopedics, Renmin Hospital of Wuhan University, Wuhan 430072, China

**Keywords:** osteoarthritis, senescence, synoviocytes, ceria

## Abstract

Accumulation of senescent cells is the prominent risk factor for osteoarthritis (OA), accelerating the progression of OA through a senescence-associated secretory phenotype (SASP). Recent studies emphasized the existence of senescent synoviocytes in OA and the therapeutic effect of removing senescent synoviocytes. Ceria nanoparticles (CeNP) have exhibited therapeutic effects in multiple age-related diseases due to their unique capability of ROS scavenging. However, the role of CeNP in OA remains unknown. Our results revealed that CeNP could inhibit the expression of senescence and SASP biomarkers in multiple passaged and hydrogen-peroxide-treated synoviocytes by removing ROS. In vivo, the concentration of ROS in the synovial tissue was remarkably suppressed after the intra-articular injection of CeNP. Likewise, CeNP reduced the expression of senescence and SASP biomarkers as determined by immunohistochemistry analysis. The mechanistic study showed that CeNP inactivated the NFκB pathway in senescent synoviocytes. Finally, safranin O–fast green staining showed milder destruction of articular cartilage in the CeNP-treated group compared with the OA group. Overall, our study suggested that CeNP attenuated senescence and protected cartilage from degeneration via scavenging ROS and inactivating the NFκB signaling pathway. This study has potentially significant implications in the field of OA as it provides a novel strategy for OA treatment.

## 1. Introduction

Osteoarthritis (OA) is the most common type of arthritis characterized by synovitis, articular cartilage degeneration, subchondral bone sclerosis and osteophyte formation [1]. About 80% of people over 65 have imaging changes in OA, affecting the quality of life of the elderly and inflicting a heavy economic burden on families and society [2]. Synovitis emerges in the early stage of OA and is associated with the symptoms and structural progression of OA [1]. In response to cartilage degradation and cytokine stimulation, synoviocytes secrete pro-inflammatory mediators, exacerbating the inflammatory responses and pain [3]. It has been reported that synovitis is positively correlated with the degree of pain [4]. In addition, matrix-degrading enzymes released by synoviocytes lead to the irreversible destruction of cartilage [5]. Thus, it is of great significance to develop the treatment for synovitis to relieve and delay the progression of age-related OA.

Cellular senescence refers to an irreversible state of cell cycle arrest, which is characterized by increased activity of senescence-associated β-galactosidase (SA-β-Gal) and a senescence-associated secretory phenotype (SASP) [6]. SASP alters the cellular microenvironment by secreting excessive pro-inflammatory mediators and matrix-degrading enzymes [7]. Senescent synoviocytes play a vital role in the progression of OA, which has gradually attracted the attention of researchers. Zhang et al. reported that high expression of the senescence marker, p16, existed in synovial tissue of OA patients [8]. Jeon et al. observed an increased proportion of cells positively staining for p16 and SA-β-Gal in the OA synovium [9]. Chen et al. proposed that targeting the senescent synoviocytes could effectively alleviate the progression of OA [10,11]. The above studies indicate that the targeted intervention on senescent synoviocytes is effective for OA treatment. 

Reactive oxygen species (ROS), including hydrogen peroxide, hydroxyl radicals, superoxide anions and nitric oxide, are unstable and highly reactive due to their unpaired electrons [12]. ROS could lead to cellular senescence. Meanwhile, ROS production was significantly increased due to mitochondrial dysfunction and the abnormal activity of NADPH oxidases in senescent cells [13]. In OA synoviocytes, excessive ROS activated nuclear factor kappa B (NFκB) pathways, promoted the synthesis of inflammatory cytokines and matrix-degrading enzymes, and led to downstream events such as synovial cartilage inflammation and cartilage matrix destruction [14,15,16]. In addition, scavenging ROS in synoviocytes could reduce the inflammatory response, inhibit the release of matrix-degrading enzymes and promote cartilage matrix synthesis [17,18,19]. Therefore, scavenging the ROS may prevent the senescence and SASP of synoviocytes, and further delay the progression of OA.

Ceria nanoparticles (CeNP) have recently attracted great attention due to their unique antioxidant capacity [20,21,22]. CeNP can mimic the activity of two key antioxidant enzymes, superoxide dismutase and catalase, abating the noxious intracellular ROS. CeNP has been shown to have therapeutic effects in ROS-related diseases such as Alzheimer’s disease, stroke, liver diseases, glaucoma and acute kidney injury [23,24,25,26,27]. However, the therapeutic effects and mechanism of CeNP on OA remain unclear. We speculated that CeNP may alleviate the SASP in senescent synoviocytes by scavenging ROS.

In this study, we provide evidence that CeNP is effective in alleviating senescence and SASP of synoviocytes in vitro and in vivo, and that CeNP may serve as a new strategy for OA treatment.

## 2. Results

### 2.1. Synthesis and Characterization of CeNP

**Figure 1:**
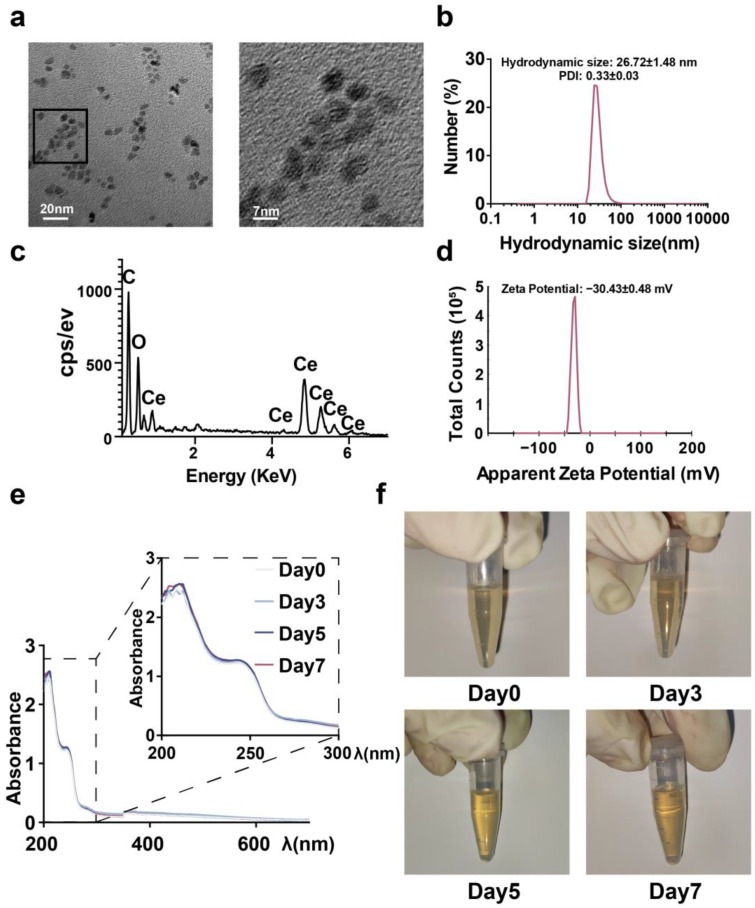
Characterization of CeNP. (**a**) Representative TEM images of CeNP and magnified views of the black box. (**b**) The hydrodynamic size of CeNP. (**c**) Representative EDS spectrum of CeNP. (**d**) The zeta potential of CeNP. UV–visible spectra (**e**) and appearance (**f**) of CeNP in aqueous solution at room temperature for one week.

The uniform-sized CeNP were synthesized using the thermal decomposition method. CeNP were transferred to the aqueous phase for the subsequent experiments by coating with mPEG_2k_-DSPE. TEM showed that CeNP were uniformly spherical with an average size of 4.74 ± 0.59 nm (Figure 1a). Due to the coated mPEG_2k_-DSPE and water shell, the hydrodynamic particle diameter was 26.72 ± 1.48 nm higher than the TEM size (Figure 1b). The EDS analysis suggested a Ce:O atomic ratio of 0.49 (Figure 1c). The zeta potential measurement showed the CeNP had a zeta potential of −30.43 ± 0.48 mV (Figure 1d). In addition, UV–visible spectra confirmed that CeNP were stable in the aqueous solution at room temperature for at least one week (Figure 1e,f). 

### 2.2. Extracellular Antioxidant Capacity of CeNP

**Figure 2:**
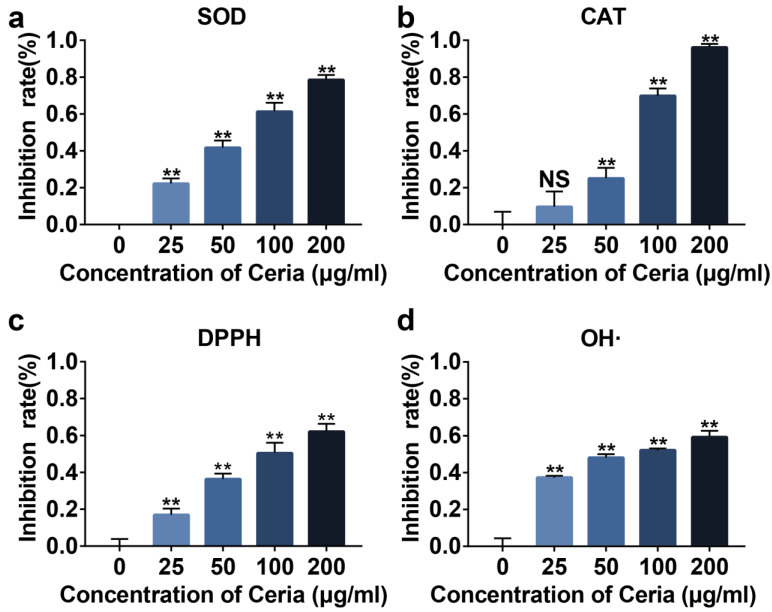
ROS scavenging capacity of CeNP. Inhibition rate of superoxide anion (**a**), hydrogen peroxide (**b**), DPPH (**c**), and OH·(**d**) in different CeNP concentrations (*n* = 3). Data represent the mean ± SEM from three independent experiments, NS, not significant; ** *p* < 0.01 vs. 0 μg/mL.

Next, we evaluated the SOD and CAT enzyme-mimetic activities conferred by Ce^3+^ and Ce^4+^ in the synthesized CeNP in vitro. The SOD and CAT activity assay showed that CeNP could reduce the concentration of superoxide anion and hydrogen peroxide in a concentration-dependent manner (Figure 2a,b, *p* < 0.01). DPPH, a stable free radical capable of absorbing hydrogen atoms from antioxidants, is used to detect free radical scavenging activity [28]. A DPPH and hydroxyl radical detection assay revealed that CeNP could decrease the concentration of free radicals in a concentration-dependent manner (Figure 2c,d, *p* < 0.01). These results suggested that CeNP possessed a great ability for ROS scavenging extracellularly.

### 2.3. Cytotoxicity and Cellular Uptake of CeNP

**Figure 3:**
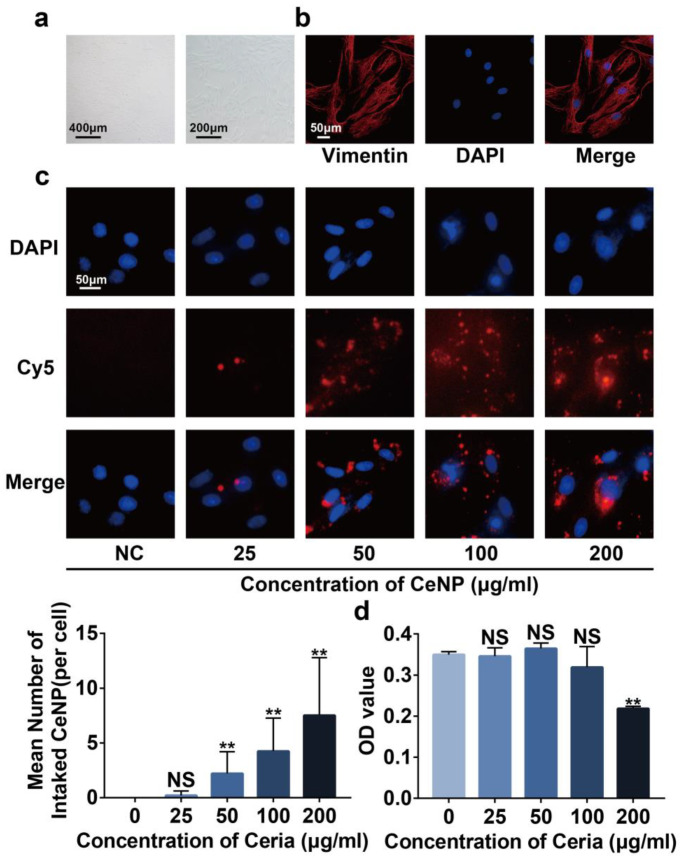
Cytotoxicity and cellular uptake of CeNP in synoviocytes. (**a**) Appearance of synoviocytes under the light microscope. (**b**) Immunofluorescence staining of vimentin in synoviocytes. (**c**) CeNP uptake capacity of synoviocytes and mean intake number of CeNP (*n* = 3). (**d**) Synoviocyte viability detected by the CCK-8 assay after treatment with different concentrations of CeNP for 24 h (*n* = 3). Data represent the mean ± SEM from three independent experiments, NS, not significant; ** *p* < 0.01 vs. NC.

We then explored the cytotoxicity of CeNP on primary rat synoviocytes. The primary synoviocytes featured as spindle-shaped under light microscopy (Figure 3a). Immunofluorescence staining showed that primary synoviocytes expressed vimentin (Figure 3b), a known marker of synoviocytes, which was consistent with previous reports [29]. Cy5 was used to label CeNP to observe the uptake of CeNP by synoviocytes. Fluorescence images indicated that Cy5-labeled CeNP dispersed around the nucleus within 24 h of co-incubation with synoviocytes, and the mean intake number of CeNP was positively related to the concentration of CeNP (Figure 3c, *p* < 0.05). The results of the CCK8 assay revealed that CeNP did not affect the cell viability with concentrations below 100 μg/mL. Still, cell viability was significantly inhibited after the concentration increased to 200 μg/mL (Figure 3d, *p* < 0.01). Combining the results above, we took 100 μg/mL as the subsequent intervention concentration and 24 h as the intervention time.

### 2.4. CeNP Attenuated H_2_O_2_-Elicited Senescence and Inhibited SASP in Synoviocytes

**Figure 4:**
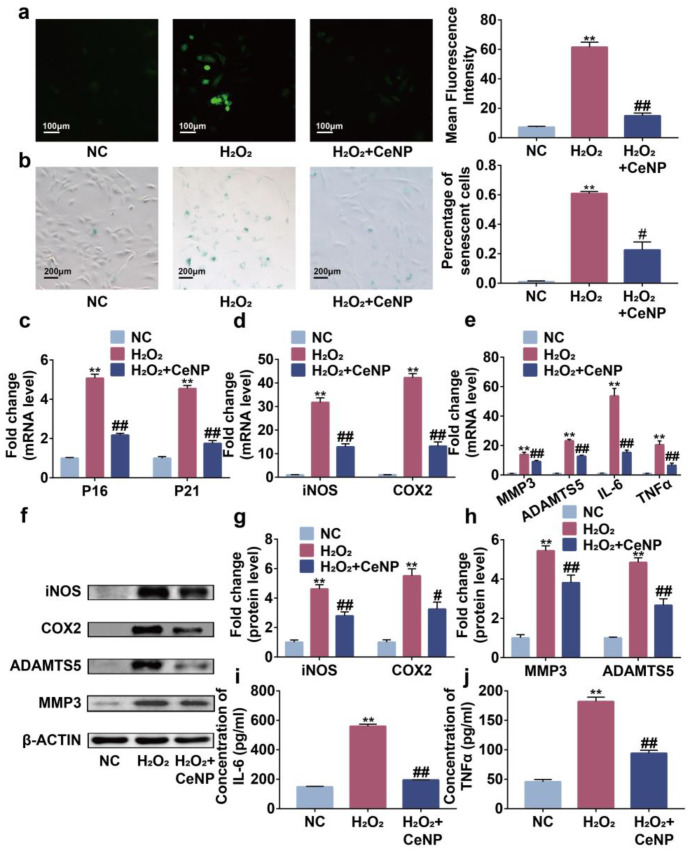
CeNP attenuated H_2_O_2_-elicited senescence and inhibited SASP in synoviocytes. (**a**) Representative fluorescence imaging of intracellular ROS and mean fluorescence intensity in NC, H_2_O_2_ and H_2_O_2_ + CeNP group (*n* = 3). (**b**) SA-β-Gal staining and quantification of SA-β-Gal positive rate in NC, H_2_O_2_ and H_2_O_2_ + CeNP groups (*n* = 3). (**c**–**e**) RT-qPCR analysis of P16, P21, iNOS, COX2, MMP3, ADAMTS5, IL-6 and TNFα in NC, H_2_O_2_ and H_2_O_2_ + CeNP groups (*n* = 3). (**f**–**h**) Western blot results of iNOS, COX2, ADAMTS5 and MMP3 in NC, H_2_O_2_ and H_2_O_2_ + CeNP groups (*n* = 3). (**i**,**j**) Elisa assays of IL-6 and TNFα in NC, H_2_O_2_ and H_2_O_2_ + CeNP groups (*n* = 3). Data represent the mean ± SEM from three independent experiments, ** *p* < 0.01 vs. NC; # *p* < 0.05 vs. H_2_O_2_ group; ## *p* < 0.01 vs. H_2_O_2_ group.

The accumulation of ROS is involved in the occurrence of senescence and SASP [30]. Accordingly, we explored the effects of CeNP in the H_2_O_2_-elicited senescence model. DCFH-DA and SA-β-Gal staining results indicated that synoviocytes possessed a higher concentration of ROS and percentage of SA-β-Gal positive cells after H_2_O_2_ treatment (Figure 4a,b, *p* < 0.01). Additionally, higher expression of P16 and P21, the senescence biomarkers, were observed in H_2_O_2_-treated synoviocytes by Rt-qPCR (Figure 4c, *p* < 0.01). These results indicated the successful construction of the senescent synoviocytes model. However, the changes induced by H_2_O_2_ were partially reversed by CeNP, revealing the effects of CeNP on attenuating senescence.

Then, to explore the role of CeNP treatment on SASP, we detected the mRNA level of SASP-related biomarkers iNOS, COX2, MMP3, ADAMTS5, IL-6 and TNFα by Rt-qPCR. We found that H_2_O_2_ promoted the expression of iNOS, COX2, MMP3, ADAMTS5, IL-6 and TNFα on mRNA levels (Figure 4d,e, *p* < 0.01). Simultaneously, western blot analysis showed that H_2_O_2_ elevated the protein levels of iNOS, COX2, MMP3 and ADAMTS5 (Figure 4f–h, *p* < 0.01). The ELISA results confirmed the increased secretion of IL-6 and TNFα on H_2_O_2_-treated synoviocytes (Figure 4i,j, *p* < 0.01). Expectedly, CeNP suppressed these alterations, indicating that CeNP could inhibit SASP in senescent synoviocytes. These results suggested that CeNP attenuated oxidative-stress-associated senescence and inhibited SASP through scavenging ROS synoviocytes.

### 2.5. CeNP Attenuated Senescence and Inhibited SASP in Multiple Passaged Synoviocytes

**Figure 5:**
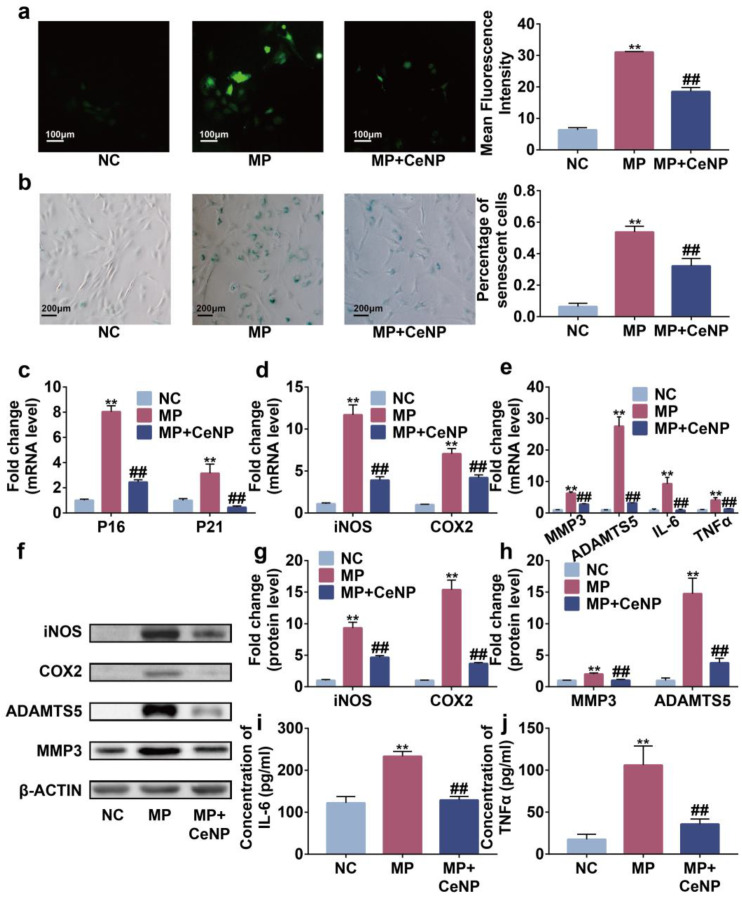
CeNP attenuated senescence and inhibited SASP in multiple passaged synoviocytes. (**a**) Representative fluorescence imaging of intracellular ROS and mean fluorescence intensity in NC, MP, and MP with CeNP treatment synoviocytes (*n* = 3). (**b**) SA-β-Gal staining and quantification of SA-β-Gal positive rate in NC, MP, and MP with CeNP treatment synoviocytes (*n* = 3). (**c**–**e**) RT-qPCR analysis of P16, P21, iNOS, COX2, MMP3, ADAMTS5, IL-6 and TNFα in NC, MP, and MP with CeNP treatment synoviocytes (*n* = 3). (**f**–**h**) Western blot results of iNOS, COX2, ADAMTS5 and MMP3 in NC, MP, and MP with CeNP treatment synoviocytes (*n* = 3). (**i**,**j**) Elisa assays of IL-6 and TNFα in NC, MP, and MP with CeNP treatment synoviocytes (*n* = 3). Data represent the mean ± SEM from three independent experiments, ** *p* < 0.01 vs. NC; ## *p* < 0.01 vs. MP.

Subsequently, we constructed a multiple passaged synoviocytes model to simulate replicative senescence occurring in synovitis of OA, and the same experiments were conducted with similar results [31,32]. CeNP abolished the accumulation of ROS and senescent synoviocytes induced by multiple passages (Figure 5a–c, *p* < 0.01). Concurrently, CeNP reduced the expression of iNOS, COX2, MMP3, ADAMTS5, IL-6 and TNFα at mRNA and protein levels (Figure 5d–j, *p* < 0.01). Therefore, CeNP also attenuated replicative senescence in synoviocytes.

### 2.6. CeNP Inhibited the Activation of NFκB Pathway in Senescent Synoviocytes

**Figure 6:**
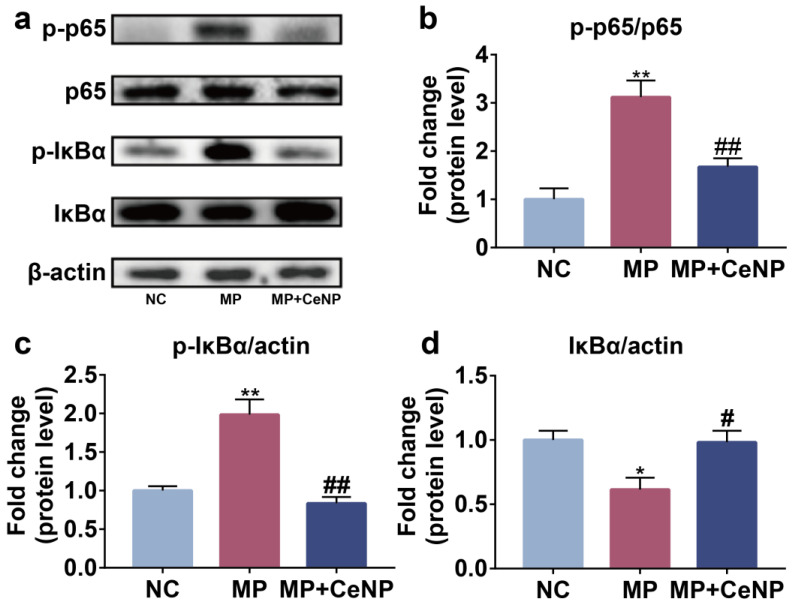
CeNP inhibited the activation of the NFκB pathway in senescent synoviocytes. (**a**) The protein levels of p-p65, p65, p-IκBα, and IκBα were detected by western blot. (**b**–**d**) Quantitative analysis of the results. * *p* < 0.05 vs. NC; ** *p* < 0.01 vs. NC; # *p* < 0.05 vs. MP; ## *p* < 0.01 vs. MP.

As previously described, excessive ROS promoted the expression of inflammatory cytokines and matrix-degrading enzymes by activating the NFκB pathway in synoviocytes [14,15,16]. Therefore, we examined the impact of CeNP on the NFκB pathway in MP synoviocytes. Western blot results showed that the expression of p-p65 and p-IκBα was elevated in MP synoviocytes compared with NC synoviocytes (Figure 6a–c, *p* < 0.01), and the expression of IκBα was decreased (Figure 6d, *p* < 0.01), while CeNP significantly abolished the increased expression of p-IκBα and p-p65 in MP synoviocytes and reduced the degradation of IκBα. These results indicated that CeNP could dramatically inhibit the activation of the NFκB pathway in senescent synoviocytes.

### 2.7. CeNP Scavenged ROS and Attenuated Senescence of Synoviocytes In Vivo

**Figure 7:**
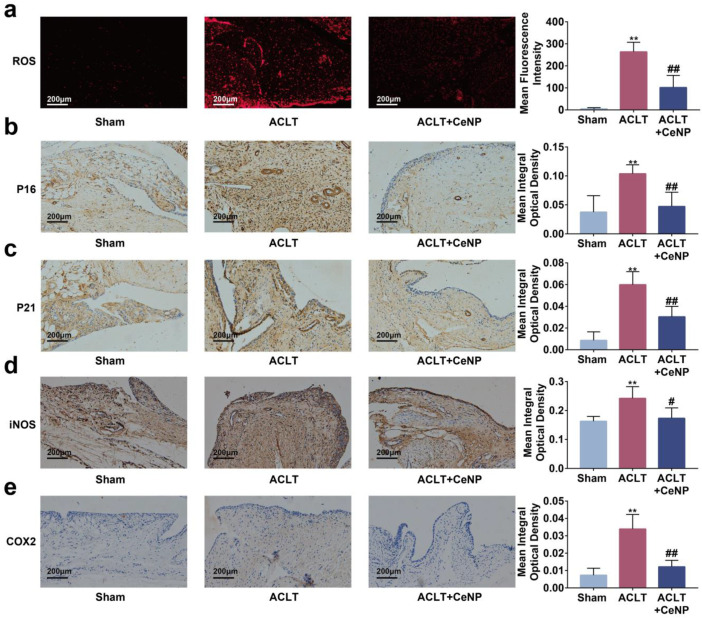
CeNP scavenged ROS and attenuated senescence of synoviocytes in vivo. (**a**) DHE staining and mean fluorescence intensity of ROS in the synovium (*n* = 5). (**b**–**e**) Immunohistochemical staining and quantitative results of P16, P21, iNOS and COX2 in the synovium (*n* = 5). ** *p* < 0.01 vs. sham; # *p* < 0.05 vs. ACLT; ## *p* < 0.01 vs. ACLT.

**Figure 8:**
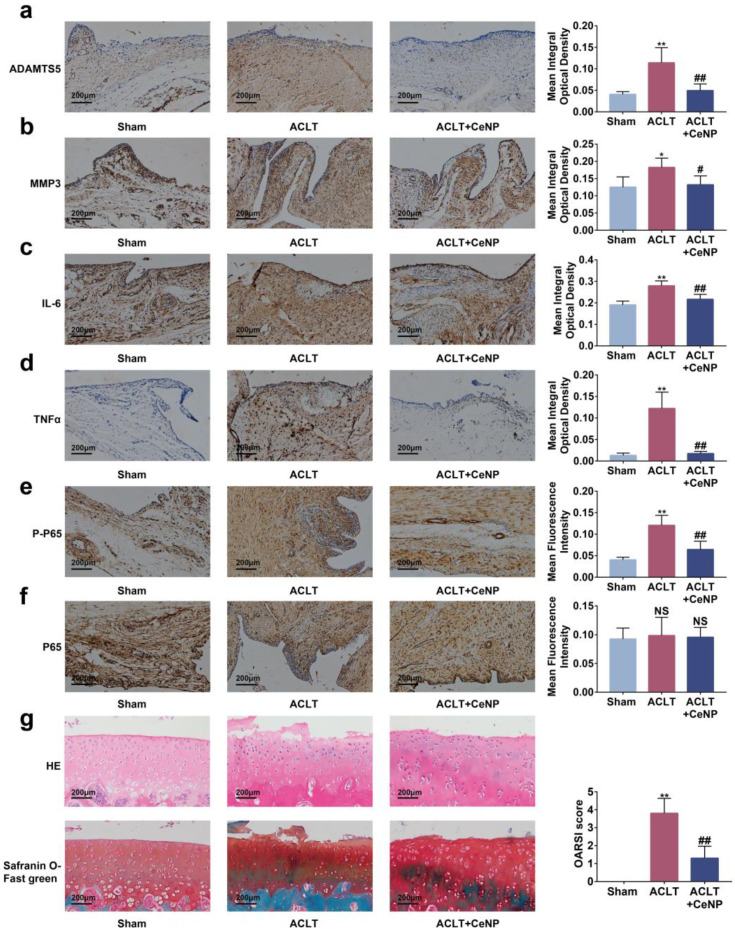
CeNP suppressed SASP, inactivated the NFκB signaling pathway and protected cartilage in vivo. (**a**–**d**) Immunohistochemical staining and quantitative results of ADAMTS5, MMP3, IL-6 and TNFα (*n* = 5). (**e**,**f**) Immunohistochemical staining and quantitative results of p65 and p-p65 (*n* = 5). (**g**) Representative pictures of HE, safranin O–fast green staining of the cartilage and OARSI score in each group (*n* = 5). * *p* < 0.05 vs. sham; ** *p* < 0.01 vs. sham; # *p* < 0.05 vs. ACLT; ## *p* < 0.01 vs. ACLT.

Previous studies have reported that senescent cells accumulated in the synovium after ACLT [9]. To manifest the effects of CeNP on senescent synoviocytes in vivo, we established a rat OA model via ACLT surgery. We found that the concentration of ROS was elevated in the synovium after ACLT, and that intra-articular injections of CeNP reduced ROS content as determined by lower fluorescence intensity in the CeNP treatment group (Figure 7a, *p* < 0.01). The immunohistochemical images and quantitative analysis showed that the intra-articular injection of CeNP inhibited the expression of P16, P21, iNOS and COX2, indicating that the senescence of synoviocytes was attenuated by CeNP (Figure 7b–e, *p* < 0.01). Likewise, the expression of SASP biomarkers ADAMTS5, MMP3, IL-6 and TNFα was suppressed after CeNP treatment in the ACLT group (Figure 8a–d, *p* < 0.01). Collectively, CeNP could attenuate the senescence and SASP via removing ROS in vivo.

### 2.8. CeNP Inactivated the NFκB Signaling Pathway and Protected Cartilage In Vivo

To assess the activation of the NFκB signaling pathway in vivo, we tested the protein levels of p65 and p-p65 on the synovium. Immunohistochemical staining and quantitative results revealed that CeNP reduced the levels of p-p65 protein (Figure 8e, *p* < 0.01). However, no significant difference was observed in the levels of p65 in each group (Figure 8f, *p* > 0.01). Finally, we performed HE and safranin O–fast green staining to evaluate the protective role of CeNP in articular cartilage. The result showed that cartilage erosions and proteoglycan loss occurred in the ACLT group, while the CeNP group exhibited more mild change and a lower OARSI score (Figure 8g, *p* < 0.01). In summary, CeNP could inactivate the NFκB signaling pathway and protected cartilage in vivo.

## 3. Discussion

Synovium is the special connective tissue wrapping the joint and is responsible for producing synovial fluid and providing nutrients to cartilage [9]. Synovial inflammation is a pathological phenomenon throughout the whole process of OA, which occurs under the stimulation of inflammatory cytokines and cartilage matrix degradation products. In this case, synoviocytes secreted pro-inflammatory mediators and matrix-degrading enzymes, and aggravated joint inflammation and cartilage degradation [3]. Histologically, synovial inflammation mainly manifested as proliferative inflammation, such as synovial lining hyperplasia, immune cell infiltration, angiogenesis and fibrosis [33]. As a result, few studies have linked synovial inflammation to synoviocytes senescence, a proliferation-suppressed phenotype. Zhang and Chen et al. recently discussed the pathogenic effects of increasing senescent synoviocytes in OA, which suggested senescent synoviocytes might accelerate the progression of OA [8,11].

Cerium is a lanthanide metal element and exists in a mixture of trivalent and tetravalent. The conversion of cerium ions between trivalent and tetravalent endows it with repeatable reducibility. Since oxygen vacancies and Ce^3+^ mostly existed on the surface, CeNP, with higher surface area to volume ratios, had better reducibility than cerium oxide with larger particles [34]. In this work, we synthesized CeNP with the size of 4.74 ± 0.59 nm, exhibiting good stability and ROS scavenging effects. CeNP have shown general safety and sound therapeutic effects in various ROS-related diseases, which attracted us to explore their therapeutic roles in OA. However, some studies reported the presence of concentration-dependent cytotoxicity of CeNP in multiple cell types [35,36]. Our results found that the cellular viability of synoviocytes exposed to excessive CeNP decreased, consistent with previous reports. This could be due to DNA damage, dephosphorylation of various substrates, aberrant cell signaling and alterations in the transcriptional and posttranslational levels induced by CeNP [37,38,39]. However, the toxicological mechanism of CeNP remains unknown and merits further study [40].

Oxidative stress was considered to be the crucial cause of senescent synoviocytes [41]. Meanwhile, oxidative stress signals in senescent cells promoted the occurrence of SASP [42]. Our study explored the role of scavenging ROS by CeNP in the MP and H_2_O_2_-induced senescent synoviocytes. We found that the concentration of ROS and percentage of SA-β-Gal positive cells increased, and SASP-related biomarkers iNOS, COX2, MMP3, ADAMTS5, IL-6 and TNFα were up-regulated in senescent synoviocytes. After CeNP treatment, the concentration of ROS and percentage of SA-β-Gal positive cells were reduced, and SASP-related biomarkers were suppressed.

The ACLT model is the most commonly used surgical model in OA research and is suitable for pharmaceutical studies because of its slow development [43]. Meanwhile, the ROS generation and senescent cell accumulation have been observed in the ACLT-induced OA model [9,44,45]. Therefore, we sought to further confirm the ROS-scavenging capacity of CeNP in ACLT-induced OA in rats. In this study, accumulated ROS and increasing expression of P16, P21, iNOS, COX2, MMP3, ADAMTS5, IL-6 and TNFα were observed in the OA synovium. After intra-articular injections of CeNP, the concentration of ROS and the protein levels of the molecules above were inhibited apparently in the synovium. In addition, the intra-articular injections of CeNP preserved proteoglycan loss in ACLT rats. Therefore, CeNP was capable of attenuating senescence in the synovium and protecting articular cartilage from deterioration via scavenging ROS.

NFκB is a family of dimeric transcription factors involved in cell differentiation, proliferation, survival, and serves the function of coordinating inflammatory responses [46]. NFκB plays a crucial role in the progression of OA. The activation of NFκB signaling led to the expression of inflammatory cytokines and matrix-degrading enzymes [47,48,49,50,51,52]. ROS activated NFκB signaling through regulating multiple NFκB signaling-related proteins such as inhibiting the phosphorylation of IκBα [53]. In this study, we found that CeNP inhibited the protein level of p-p65 and p-IκBα in multiple passaged synoviocytes, and attenuated the degradation of IκBα. Furthermore, the relative expression of p-p65 was down-regulated in OA rats after CeNP injection, demonstrating that CeNP could inhibit the NFκB pathway activity in senescent synoviocytes through ROS scavenging, thereby inhibiting the SASP.

There are some limitations to this study. We failed to measure the concentration of intra-articular SASP protein due to less synovial fluid in rats. For ethical reasons, we were unable to obtain normal synoviocytes for the experiment. Moreover, OA is a whole-joint disease, and the effects of CeNP in other tissues such as chondrocytes or immune cells need to be investigated.

## 4. Materials and Methods

### 4.1. Materials

Cerium nitrate hexahydrate (Ce(NO_3_)_3_·6H_2_O), oleylamine, 1-octadecene (ODE), acetone, methanol, cyclohexane and anhydrous were purchased from Aladdim (Shanghai, China). NH_2_-PEG_2k_-DSPE, mPEG_2k_-DSPE and Cy5-NHS were acquired from Yuanye Bio-Technology (Shanghai, China).

### 4.2. Synthesis of CeNP

CeNP was synthesized according to previously reported methods [54]. Briefly, Ce(NO_3_)_3_·6H_2_O (1.736 g, 4 mmol) and oleylamine (3.208 g, 12 mmol) were dispersed in 20 g of ODE. The mixed solution was stirred at room temperature for 2 h and then heated under a vacuum at 80 °C for 1 h to remove water. The mixture was heated and maintained at 260 °C for 2 h in an argon atmosphere. After being cooled to room temperature, acetone and methanol were added to the mixture to precipitate CeNP. CeNP were washed with cyclohexane and anhydrous ethanol and collected by centrifugation at 15,000 rpm for 20 min. This washing process was repeated five times. Finally, CeNP were dispersed in chloroform.

The CeNP were decorated with mPEG_2k_-DSPE through ultrasound for transferring into the aqueous phase. Then, mPEG_2k_-DSPE (30 mg) were added into 5 mL of CeNP/chloroform (2 mg/mL), and stirred at room temperature for 4 h. After evaporating the chloroform by rotary evaporation, mPEG_2k_-DSPE-modified CeNP were dispersed in 10 mL of ultrapure water by ultrasonic water bath for 30 min. CeNP were purified by filtration, ultracentrifugation and dialysis (MW cutoff = 8–14 kDa). An inductively coupled plasma optical emission spectrometer was used to calculate the molality of CeNP.

For the synthesis of Cy5-labeled CeNP, Cy5-NHS (5 mg) and NH_2_-PEG_2k_-DSPE (20 mg) were dissolved in 1 mL of DMSO, and the mixture was stirred overnight at room temperature under Ar protection. Cy5-PEG_2k_-DSPE was purified by dialysis (MW cutoff = 500–1000 Da) and lyophilized for 24 h. Cy5-PEG_2k_-DSPE (5 mg) and mPEG_2k_-DSPE (25 mg) were added to 5 mL of CeNP/chloroform (2 mg/mL) and the above procedures repeated to obtain Cy5-labeled CeNP.

### 4.3. Characterization of CeNP

Morphology and elemental distribution of CeNP were detected by the transmission electron microscopy (TEM) and the energy dispersive spectrometer in JEM-2100 TEM (JEOL, Tokyo, Japan). The zeta potentials and particle diameters of the CeNP were measured by light scattering in Malvern Zetasizer Nano Series (Malvern, UK).

### 4.4. Extracellular Antioxidant Capacity of CeNP

The antioxidant capacity of CeNP was assessed by multiple assays according to the manufacturer’s protocols. The superoxide dismutase (SOD) assay kit (Nanjinjiancheng, Nanjin, China, A001-3-2) and catalase (CAT) assay kit (Nanjinjiancheng, A007-1-1) were used to assess the SOD and CAT enzyme activities of CeNP. The free radical scavenging effect of CeNP was tested by hydroxyl free radical assay kit (Nanjinjiancheng, A018-1-1) and 2,2-Diphenyl-1-picrylhydrazyl (DPPH) free radical scavenging capacity assay kit (Nanjinjiancheng, A153-1-1).

### 4.5. Cell Isolated and Cultured

We isolated synovial tissues from Wistar rats (8 weeks, males) to isolate the primary synoviocytes. After mincing, synovial tissues were placed into DMEM/F12 medium with 0.2% collagenase II for 4 h at 37 °C. Synoviocytes were collected by filtration and centrifugation and maintained in DMEM/F12 medium supplemented with 10% fetal bovine serum (Gibco, NY, America, 10270-106) and 100 U/mL of penicillin–streptomycin (Servicebio, Wuhan, China, G4003) in an incubator at 37 °C with 5% CO_2_. For the multiple passaged senescent synoviocytes model, synoviocytes were subcultured for 6–8 passages till the growth rate decreased significantly [55]. For the H_2_O_2_-elicited senescent synoviocytes model, synoviocytes were treated with 500 μM H_2_O_2_(Aladdin, Shanghai, China, 7722-84-1) for 4 h [56].

### 4.6. Cellular Uptake of CeNP

Synoviocytes were treated with 100 mg/mL Cy5-labeled CeNP for 24 h and washed three times with PBS. After being fixed with 4% paraformaldehyde for 15 min, nuclei were stained with DAPI. The fluorescence images were visualized using the IX73 inverted fluorescence microscope (OLYMPUS, Tokyo, Japan).

### 4.7. Cytotoxicity Assessment

To measure the cytotoxicity of CeNP, we seeded 5000 synoviocytes per well in a 96-well plate. After adherence, the medium was replaced by the fresh medium with different CeNP concentrations (0, 25, 50, 100, 200 mg/mL), and cultured at 37 °C for 24 h [23]. Next, 10 μL of Cell Counting Kit-8 (CCK-8, Servicebio, G4103) reagent was added to each well and incubated at 37 °C for 2 h. The absorbance at 450 nm was determined using a Microplate Reader (EnVision, PerkinElmer, MA, America).

### 4.8. ROS Assay

Intracellular ROS was measured by DCFH-DA (Beyotime, Shanghai, China, S0033S) according to the manufacturer’s protocol. Synoviocytes were loaded with DCFH-DA by incubating with 10 μM of probe solution for 30 min at 37 °C. Then, the images were visualized by the IX73 inverted fluorescence microscope, and the mean fluorescence intensity was calculated by ImageJ (versions: V1.8.0).

### 4.9. SA-β-Gal Staining

SA-β-Gal staining was performed by a SA-β-Gal staining kit (Servicebio, G1073) according to the manufacturer’s protocol. Synoviocytes were fixed with senescent cell staining fixative for 15 min. After washing three times, synoviocytes were incubated in SA-β-Gal staining solution at 37 °C for 24 h. The inverted fluorescence microscope was used to capture the images. SA-β-Gal positive synoviocytes were counted in three random fields per dish.

### 4.10. Real-Time Quantitative PCR (RT-qPCR)

The total cellular RNA was extracted by the RNA isolation kit (Beyotime, Shanghai, China, R0026) according to the manufacturer’s instructions. The RNA concentrations were measured by Nanodrop (Thermo, MA, America). Approximately 1000 ng of RNA was reverse-transcripted to cDNA using a SweScript RT II Enzyme Mix (Servicebio, Wuhan, China, G3330), and Rt-qPCR was performed using the 2 × Universal SYBR Green Fast qPCR Mix (Abclonal, Wuhan, China, RK21203) on LightCycler 480 (Roche Diagnostics, Basel, Switzerland). The fold change of targets was analyzed by the 2^−ΔΔCt^ method, and β-actin was considered the internal reference. The primers used in this study are shown in Table S1.

### 4.11. Western Blot

The total proteins from synoviocytes were extracted using RIPA buffer (Servicebio, G2008), to which PMSF (Servicebio, G2008), phosphatase inhibitors (Servicebio, G2007) and 50×Cocktail (Servicebio, G2006) were added. Synoviocytes lysis was performed for 20 min on ice, and ultrasonication and centrifugation were used to purify proteins. The concentration of proteins was measured by the BCA protein assay kit (Servicebio, G2026) according to the manufacturer’s instructions. Protein samples were reduced in SDS sample buffer, and were separated by 10% SDS-PAGE. After transferring to PVDF membranes and blocking, the membranes were incubated with primary antibodies against β-actin (Servicebio, GB11001, 1:2000), iNOS (Abclonal, A3774, 1:2000), COX2 (Abclonal, A3560, 1:2000), ADAMTS5 (Abclonal, A2836, 1:2000), MMP3 (Abclonal, A11418, 1:2000), p65 (Abmart, Shanghai, China, TA5006, 1:2000), p-p65 (Abmart, TP56367, 1:2000), IkBα (Abmart, TA5002, 1:2000) and p-IkBα (Abmart, TA2002, 1:2000) overnight at 4 °C. Next, the membranes were washed three times with PBS and incubated with HRP-conjugated secondary antibodies (Servicebio, GB23303, 1:5000) for 1 h at room temperature. The ECL substrate (Epizyme Biotech, Shanghai, China, SQ101) was used to visualize the protein bands on ChemiDoc Touch (Bio-Rad, CA, America), and the semi-quantitative analysis of images was conducted using ImageJ software (versions: 1.8.0).

### 4.12. Enzyme-Linked Immunosorbent Assay (ELISA)

The concentrations of TNFα and IL-6 in synoviocyte culture media were assessed using rat ELISA (Thermo, 88-7340-88 and 88-50625-88) kits according to the manufacturer’s protocols.

### 4.13. OA Rat Model

Animal care and experimentation were consistent with the National Research Council’s Guide for the Care and Use of Laboratory Animals, and were approved by the Laboratory Animal Welfare & Ethics Committee of the Renmin Hospital of Wuhan University (Approval No: 20220103A). Fifteen 8-week-old male Wistar rats were provided by SiPeiFu Biotechnology Co. Ltd. (Beijing, China). OA was induced by anterior cruciate ligament transection (ACLT), as reported previously [57]. The joint cavity was exposed in the sham group, but the anterior cruciate ligament was not transected. After surgery, rats in the OA group and CeNP group accepted an intra-articular injection of CeNP (100 μg/mL, 50 uL) and saline (50 uL) once a week. Eight weeks post-surgery, all rats were sacrificed. The knee joint samples were fixed with 4% paraformaldehyde, decalcified in 0.5 M EDTA, and embedded in paraffin. A part of synovium tissue was fresh frozen in OCT (Servicebio, G6059) for frozen sections, and the ROS was detected by dihydroethidium (Beyotime, S0063).

### 4.14. Histology

The paraffin-embedded tissue was cut into 6 μm thick sections using a rotary microtome (Leica RM2016, Leica Microsystems Ltd., Weztlar, Germany). Then, the midsagittal sections were stained with safranin O–fast green. Microscope images were acquired with an inverted microscope (NIKON ECLIPSE CI, NIKON, Tokyo, Japan). The Osteoarthritis Research Society International (OARSI) system was used to evaluate the OA severity with two blinded pathologists [58].

### 4.15. Immunohistochemistry

The sections were subjected to antigen retrieval in citric acid buffer (Powerfor Biologu, Wuhan, China, B0034). Nonspecific protein binding was blocked by BSA (Solarbio, Beijing, China, A8010) in PBST for 30 min. Next, the sections were incubated with primary antibodies against P16 (Wanleibio, Shenyang, China, WL01418, 1:300), P21 (Wanleibio, WL0362, 1:400), iNOS (Servicebio, GB11119, 1:500), COX2 (Abclonal, A3560, 1:200), ADAMTS5 (Abclonal, A2836, 1:500), MMP3 (Abclonal, A11418, 1:200), IL-6 (Abclonal, A21264, 1:200), TNFα (Abclonal, A11534, 1:200), p65 (Abmart, TA5006, 1:50) and p-p65 (Abmart, TP56367, 1:50) overnight at 4 °C. After washing with PBS, the sections were incubated with goat anti-rabbit IgG HRP (Abcam, MA, America, ab205718,) at 37 °C for 50 min. A DAB HRP substrate kit (DAKO, Shanghai, China, K3468) was used for dye development, and hematoxylin was used as a nuclear counterstain. Microscope images were acquired with an inverted microscope (NIKON ECLIPSE CI, NIKON). Image Pro Plus (versions: 6.0) was used to quantify the results by measuring the mean integral optical density.

### 4.16. Statistics

All data in this study were reported as means ± standard error of mean (SEM). The Shapiro–Wilk normality test was used to perform the normality test. For the data with normal distribution, a one-way analysis of variance (ANOVA) followed by Bonferroni’s test was administrated. For the data with non-normal distribution, we performed the Kruskal–Wallis H test, followed by Dunn’s test. A value of *p* < 0.05 was considered significant. All reported *p* values are calculated from two-sided comparisons.

## 5. Conclusions

In summary, our study is the first, to our knowledge, to explore the therapeutic roles of CeNP in OA. We confirmed that CeNP attenuate synoviocyte senescence and SASP by clearing ROS and inactivating the NFκB pathway, which will provide a novel approach to the treatment of OA.

## Data Availability

The data presented in this study are available in the article or Supplementary Material.

## Supplementary Materials

The following supporting information can be downloaded at: https://www.mdpi.com/article/10.3390/ijms24055056/s1.

## References

[1] Goldring M.B., Otero M. (2011). Inflammation in osteoarthritis. Curr. Opin. Rheumatol..

[2] Felson D.T., Zhang Y.Q., Hannan M.T., Naimark A., Weissman B.N., Aliabadi P., Levy D. (1995). The Incidence and Natural-History of Knee Osteoarthritis in the Elderly—The Framingham Osteoarthritis Study. Arthritis Rheum..

[3] Mathiessen A., Conaghan P.G. (2017). Synovitis in osteoarthritis: Current understanding with therapeutic implications. Arthritis Res. Ther..

[4] Neogi T., Guermazi A., Roemer F., Nevitt M.C., Scholz J., Arendt-Nielsen L., Woolf C., Niu J., Bradley L.A., Quinn E. (2016). Association of Joint Inflammation with Pain Sensitization in Knee Osteoarthritis: The Multicenter Osteoarthritis Study. Arthritis Rheumatol..

[5] Siebuhr A.S., Bay-Jensen A.C., Jordan J.M., Kjelgaard-Petersen C.F., Christiansen C., Abramson S.B., Attur M., Berenbaum F., Kraus V., Karsdal M.A. (2016). Inflammation (or synovitis)-driven osteoarthritis: An opportunity for personalizing prognosis and treatment?. Scand. J. Rheumatol..

[6] Freund A., Orjalo A.V., Desprez P.Y., Campisi J. (2010). Inflammatory networks during cellular senescence: Causes and consequences. Trends Mol. Med..

[7] Greene M.A., Loeser R.F. (2015). Aging-related inflammation in osteoarthritis. Osteoarthr. Cartil..

[8] Zhang Y.B., Zhou S.Q., Cai W.S., Han G.T., Li J.P., Chen M., Li H.H. (2020). Hypoxia/reoxygenation activates the JNK pathway and accelerates synovial senescence. Mol. Med. Rep..

[9] Jeon O.H., Kim C., Laberge R.M., Demaria M., Rathod S., Vasserot A.P., Chung J.W., Kim D.H., Poon Y., David N. (2017). Local clearance of senescent cells attenuates the development of post-traumatic osteoarthritis and creates a pro-regenerative environment. Nat. Med..

[10] Chen X., Zhang L., Shao X.Y., Gong W., Shi T.S., Dong J., Shi Y., Shen S.Y., He Y., Qin J.H. (2022). Specific Clearance of Senescent Synoviocytes Suppresses the Development of Osteoarthritis based on Aptamer-Functionalized Targeted Drug Delivery System. Adv. Funct. Mater..

[11] Chen X., Gong W., Shao X.Y., Shi T.S., Zhang L., Dong J., Shi Y., Shen S.Y., Qin J.H., Jiang Q. (2022). METTL3-mediated m(6)A modification of ATG7 regulates autophagy-GATA4 axis to promote cellular senescence and osteoarthritis progression. Ann. Rheum. Dis..

[12] Zorov D.B., Juhaszova M., Sollott S.J. (2014). Mitochondrial Reactive Oxygen Species (Ros) and Ros-Induced Ros Release. Physiol. Rev..

[13] Hernandez-Segura A., Nehme J., Demaria M. (2018). Hallmarks of Cellular Senescence. Trends Cell Biol..

[14] Son Y., Kim S., Chung H.T., Pae H.O. (2013). Reactive Oxygen Species in the Activation of MAP Kinases. Hydrog. Peroxide Cell Signal. Pt C.

[15] Thalhamer T., McGrath M.A., Harnett M.M. (2008). MAPKs and their relevance to arthritis and inflammation. Rheumatology.

[16] Ansari M.Y., Ahmad N., Haqqi T.M. (2020). Oxidative stress and inflammation in osteoarthritis pathogenesis: Role of polyphenols. Biomed. Pharmacother..

[17] Khan N.M., Haseeb A., Ansari M.Y., Devarapalli P., Haynie S., Haqqi T.M. (2017). Wogonin, a plant derived small molecule, exerts potent anti-inflammatory and chondroprotective effects through the activation of ROS/ERK/Nrf2 signaling pathways in human Osteoarthritis chondrocytes. Free Radic. Biol. Med..

[18] Arra M., Swarnkar G., Ke K., Otero J.E., Ying J., Duan X., Maruyama T., Rai M.F., O’Keefe R.J., Mbalaviele G. (2020). LDHA-mediated ROS generation in chondrocytes is a potential therapeutic target for osteoarthritis. Nat. Commun..

[19] Yang G.L., Chang C.C., Yang Y.W., Yuan L., Xu L.S.Y., Ho C.T., Li S.M. (2018). Resveratrol Alleviates Rheumatoid Arthritis via Reducing ROS and Inflammation, Inhibiting MAPK Signaling Pathways, and Suppressing Angiogenesis. J. Agric. Food Chem..

[20] Lord M.S., Berret J.F., Singh S., Vinu A., Karakoti A.S. (2021). Redox Active Cerium Oxide Nanoparticles: Current Status and Burning Issues. Small.

[21] Barker E., Shepherd J., Asencio I.O. (2022). The Use of Cerium Compounds as Antimicrobials for Biomedical Applications. Molecules.

[22] Nguyen D.D., Lai J.Y. (2022). Synthesis, bioactive properties, and biomedical applications of intrinsically therapeutic nanoparticles for disease treatment. Chem. Eng. J..

[23] Yu H., Jin F.Y., Liu D., Shu G.F., Wang X.J., Qi J., Sun M.C., Yang P., Jiang S.P., Ying X.Y. (2020). ROS-responsive nano-drug delivery system combining mitochondria-targeting ceria nanoparticles with atorvastatin for acute kidney injury. Theranostics.

[24] Kim C.K., Kim T., Choi I.Y., Soh M., Kim D., Kim Y.J., Jang H., Yang H.S., Kim J.Y., Park H.K. (2012). Ceria Nanoparticles that can Protect against Ischemic Stroke. Angew. Chem.-Int. Ed..

[25] Kwon H.J., Cha M.Y., Kim D., Kim D.K., Soh M., Shin K., Hyeon T., Mook-Jung I. (2016). Mitochondria-Targeting Ceria Nanoparticles as Antioxidants for Alzheimer’s Disease. ACS Nano.

[26] Casals G., Perramon M., Casals E., Portoles I., Fernandez-Varo G., Morales-Ruiz M., Puntes V., Jimenez W. (2021). Cerium Oxide Nanoparticles: A New Therapeutic Tool in Liver Diseases. Antioxidants.

[27] Luo L.J., Nguyen D.D., Lai J.Y. (2021). Harnessing the tunable cavity of nanoceria for enhancing Y-27632-mediated alleviation of ocular hypertension. Theranostics.

[28] Foti M.C. (2015). Use and Abuse of the DPPH center dot Radical. J. Agric. Food Chem..

[29] Qi W.Z., Lin C.X., Fan K., Chen Z.Y., Liu L.L., Feng X.F., Zhang H.Y., Shao Y., Fang H., Zhao C. (2019). Hesperidin inhibits synovial cell inflammation and macrophage polarization through suppression of the PI3K/AKT pathway in complete Freund’s adjuvant-induced arthritis in mice. Chem. Biol. Interact..

[30] Davalli P., Mitic T., Caporali A., Lauriola A., D’Arca D. (2016). ROS, Cell Senescence, and Novel Molecular Mechanisms in Aging and Age-Related Diseases. Oxid. Med. Cell. Longev..

[31] Xu C., Wang L., Fozouni P., Evjen G., Chandra V., Jiang J., Lu C., Nicastri M., Bretz C., Winkler J.D. (2020). SIRT1 is downregulated by autophagy in senescence and ageing. Nat. Cell Biol..

[32] Pazolli E., Luo X., Brehm S., Carbery K., Chung J.J., Prior J.L., Doherty J., Demehri S., Salavaggione L., Piwnica-Worms D. (2009). Senescent stromal-derived osteopontin promotes preneoplastic cell growth. Cancer Res..

[33] Scanzello C.R., Goldring S.R. (2012). The role of synovitis in osteoarthritis pathogenesis. Bone.

[34] Celardo I., Pedersen J.Z., Traversa E., Ghibelli L. (2011). Pharmacological potential of cerium oxide nanoparticles. Nanoscale.

[35] Lin W., Huang Y.W., Zhou X.D., Ma Y. (2006). Toxicity of cerium oxide nanoparticles in human lung cancer cells. Int. J. Toxicol..

[36] De Marzi L., Monaco A., De Lapuente J., Ramos D., Borras M., Di Gioacchino M., Santucci S., Poma A. (2013). Cytotoxicity and genotoxicity of ceria nanoparticles on different cell lines in vitro. Int. J. Mol. Sci..

[37] Park E.J., Choi J., Park Y.K., Park K. (2008). Oxidative stress induced by cerium oxide nanoparticles in cultured BEAS-2B cells. Toxicology.

[38] Hussain S., Al-Nsour F., Rice A.B., Marshburn J., Yingling B., Ji Z., Zink J.I., Walker N.J., Garantziotis S. (2012). Cerium dioxide nanoparticles induce apoptosis and autophagy in human peripheral blood monocytes. ACS Nano.

[39] Cheng G., Guo W., Han L., Chen E., Kong L., Wang L., Ai W., Song N., Li H., Chen H. (2013). Cerium oxide nanoparticles induce cytotoxicity in human hepatoma SMMC-7721 cells via oxidative stress and the activation of MAPK signaling pathways. Toxicol. In Vitro.

[40] Yadav N. (2022). Cerium oxide nanostructures: Properties, biomedical applications and surface coatings. 3 Biotech.

[41] Wu C.J., Liu R.X., Huan S.W., Tang W., Zeng Y.K., Zhang J.C., Yang J., Li Z.Y., Zhou Y., Zha Z.G. (2022). Senescent skeletal cells cross-talk with synovial cells plays a key role in the pathogenesis of osteoarthritis. Arthritis Res. Ther..

[42] Wei W., Ji S. (2018). Cellular senescence: Molecular mechanisms and pathogenicity. J. Cell. Physiol..

[43] Kuyinu E.L., Narayanan G., Nair L.S., Laurencin C.T. (2016). Animal models of osteoarthritis: Classification, update, and measurement of outcomes. J. Orthop. Surg. Res..

[44] Kim J.E., Song D.H., Kim S.H., Jung Y., Kim S.J. (2018). Development and characterization of various osteoarthritis models for tissue engineering. PLoS ONE.

[45] Kumar S., Adjei I.M., Brown S.B., Liseth O., Sharma B. (2019). Manganese dioxide nanoparticles protect cartilage from inflammation-induced oxidative stress. Biomaterials.

[46] Oeckinghaus A., Ghosh S. (2009). The NF-kappaB family of transcription factors and its regulation. Cold Spring Harb. Perspect. Biol..

[47] Xian-Peng G., Can Y.H., Zhang C.G., Zhou C.Y., Ma K.T., Meng J.H., Ma X.C. (2011). Requirement of the NF-κB pathway for induction of Wnt-5A by interleukin-1β in condylar chondrocytes of the temporomandibular joint: Functional crosstalk between the Wnt-5A and NF-κB signaling pathways. Osteoarthr. Cartil..

[48] Goldring M.B., Marcu K.B. (2009). Cartilage homeostasis in health and rheumatic diseases. Arthritis Res. Ther..

[49] Shakibaei M., John T., Schulze-Tanzil G., Lehmann I., Mobasheri A. (2007). Suppression of NF-κB activation by curcumin leads to inhibition of expression of cyclo-oxygenase-2 and matrix metalloproteinase-9 in human articular chondrocytes: Implications for the treatment of osteoarthritis. Biochem. Pharmacol..

[50] Kapoor M., Martel-Pelletier J., Lajeunesse D., Pelletier J.P., Fahmi H. (2011). Role of proinflammatory cytokines in the pathophysiology of osteoarthritis. Nat. Rev. Rheumatol..

[51] Knobloch T.J., Madhavan S., Nam J., Agarwal S., Agarwal S. (2008). Regulation of chondrocytic gene expression by biomechanical signals. Crit. Rev. Eukaryot. Gene Expr..

[52] Liao S.Y., Zhou K., Li D.Q., Xie X.M., Jun F., Wang J. (2016). Schisantherin A suppresses interleukin-1β-induced inflammation in human chondrocytes via inhibition of NF-κB and MAPKs activation. Eur. J. Pharmacol..

[53] Zhang J., Wang X., Vikash V., Ye Q., Wu D., Liu Y., Dong W. (2016). ROS and ROS-Mediated Cellular Signaling. Oxid. Med. Cell. Longev..

[54] Lee S.S., Zhu H.G., Contreras E.Q., Prakash A., Puppala H.L., Colvin V.L. (2012). High Temperature Decomposition of Cerium Precursors to Form Ceria Nanocrystal Libraries for Biological Applications. Chem. Mater..

[55] Kang D., Shin J., Cho Y., Kim H.S., Gu Y.R., Kim H., You K.T., Chang M.J., Chang C.B., Kang S.B. (2019). Stress-activated miR-204 governs senescent phenotypes of chondrocytes to promote osteoarthritis development. Sci. Transl. Med..

[56] Liu R.F., Hu L., Wu J.N., Wang J.X., Wang X.Y., Liu Z.Y., Zhao Q.D., Li W.J., Song X.D., Xiao J.H. (2022). Changes in tumor suppressors and inflammatory responses during hydrogen peroxide-induced senescence in rat fibroblasts. Free Radic. Res..

[57] Zhou P.H., Qiu B., Denga R.H., Li H.J., Xu X.F., Shang X.F. (2018). Chondroprotective Effects of Hyaluronic Acid-Chitosan Nanoparticles Containing Plasmid DNA Encoding Cytokine Response Modifier A in a Rat Knee Osteoarthritis Model. Cell. Physiol. Biochem..

[58] Glasson S.S., Chambers M.G., Van Den Berg W.B., Little C.B. (2010). The OARSI histopathology initiative—Recommendations for histological assessments of osteoarthritis in the mouse. Osteoarthr. Cartil..

