# Supplementary material for: Ceria Nanoparticles Alleviated Osteoarthritis through Attenuating Senescence and Senescence-Associated Secretory Phenotype in Synoviocytes
Source: Int J Mol Sci. 2023 Mar 6;24(5):5056. doi: 10.3390/ijms24055056 (PMC10003033; doi:10.3390/ijms24055056)
Supplement: Supplementary file 1 [file ijms-24-05056-s001.zip › ijms-2216735-supplementary.pdf]

Supplementary material

Table S1. Primer sequences used in this study.

Gene name	F/R	Sequences 5'-3'
β-actin	F	TGCTATGTTGCCCTAGACTTCG
	R	GTTGGCATAGAGGTCTTTACGG
p16	F	GAGGACCCCACCACCCTCTC
	R	ATACCGCAAATACCGCACGA
p21	F	AGCAGTTGAGCCGCGATTG
	R	ACCCAGGGCTCAGGTAGATCTTG
TNFα	F	TCCAGAACTCCAGGCGGT
	R	TTGGTGGTTTGCTACGAC
IL-6	F	AAATCTGCTCTGGTCTTC
	R	AGGGTTTCAGTATTGCTC
MMP3	F	CATGAACTTGGCCACTCCCT
	R	TGGGTACCACGAGGACATCA
ADAMTS5	F	ACAAGAGTCTGGAGGTGAGCAAG
	R	ACATATGGTCCCAACGTCTGC
iNOS	F	CAGGCTGGAAGCCGTAACAA
	R	CGATGCACAACTGGGTGAAC
COX2	F	GTTCCAACCCATGTCAAAACCGT
	R	GGCCCTGGTGTAGTAGGAGAGGT
